# Dr. Flanner

**Published:** 1833

**Authors:** 


Thomas Flanner, M. D: of *Zanesville, O.* The deceased was a native of Pennsylvania. He graduated in the University of that state, about the year 1818, and was for 12 months a house surgeon in the Pennsylvania Hospital. Emigrating to Ohio, after this course of clinical studies, he first settled in St. Clairsville, but afterwards removed to Zanesville, where he soon earned a high reputation. At the time of his death, he was President of the General Medical Society of the state. When the Cholera appeared in Wheeling, in the month of May, he determined to visit that place, and study its character before it should reach the people who might look to him for advice. On his way back, he was seized with the disease, and died before he reached home.

